# A Novel Anticancer Therapy That Simultaneously Targets Aberrant p53 and Notch Activities in Tumors

**DOI:** 10.1371/journal.pone.0046627

**Published:** 2012-10-10

**Authors:** Yuting Yao, Li Wang, He Zhang, Haibo Wang, Xiaoping Zhao, Yidan Zhang, Leilei Zhang, Xianqun Fan, Guanxiang Qian, Ji-Fan Hu, Shengfang Ge

**Affiliations:** 1 Ninth People’s Hospital, Shanghai Jiaotong University School of Medicine, Shanghai, People’s Republic of China; 2 VA Palo Alto Health Care System, Stanford University Medical School, Palo Alto, California, United States of America; 3 Department of Biochemistry and Molecular Biology, Shanghai Jiao Tong University School of Medicine, Shanghai, People’s Republic of China; Wayne State University School of Medicine, United States of America

## Abstract

Notch signaling pathway plays an important role in tumorigenesis by maintaining the activity of self-renewal of cancer stem cells, and therefore, it is hypothesized that interference of Notch signaling may inhibit tumor formation and progression. H101 is a recombinant oncolytic adenovirus that is cytolytic in cells lacking intact p53, but it is unable to eradicate caner stem cells. In this study, we tested a new strategy of tumor gene therapy by combining a Notch1-siRNA with H101 oncolytic adenovirus. In HeLa-S3 tumor cells, the combined therapy blocked the Notch pathway and induced apoptosis in tumors that are p53-inactive. In nude mice bearing xenograft tumors derived from HeLa-S3 cells, the combination of H101/Notch1-siRNA therapies inhibited tumor growth. Moreover, Notch1-siRNA increased Hexon gene expression at both the transcriptional and the translational levels, and promoted H101 replication in tumors, thereby enhancing the oncolytic activity of H101. These data demonstrate the feasibility to combine H101 p53-targted oncolysis and anti-Notch siRNA activities as a novel anti-cancer therapy.

## Introduction

Most forms of cancer chemotherapy are unable to eradicate all malignant cells, and they often are highly toxic because of their lack of selectivity to cancer cells. As a result, new efforts have focused on developing interventions that include tumor-specific replicating viruses and siRNA.

A virus-based strategy takes advantage of the fact that the intracellular replication and production of adenoviral progeny requires the cell cycle gatekeeper p53 to be in an inactive status, and in many tumors, p53 is either mutated or epigenetically silenced. The viral early gene *E1B*, which encodes a 55-kDa protein (*E1B* 55K), is essential to viral replication. *E1B* interacts with cellular *p53* and inactivates it to allow viral replication. ONYX-015, a modified adenovirus lacking the *E1B* 55K gene, can only replicate and lyse tumor cells that have inactivated *p53*, sparing the normal cells that retain wild-type *p53* function [1]. Clinical trials in patients with recurrent head and neck cancer, metastatic colorectal cancer, or pancreatic cancer have shown that ONYX-015, when used alone or in combination with chemotherapy, is safe and has significant antitumor activity in a subset of patients [2], [3], [4].

In China, an oncolytic adenovirus called H101 has been clinically approved for the treatment of several malignancies [5]. This virus selectively infects and kills only those cells that lack active p53 viral oncolysis because the viral proteins E1B and E3 are deleted [6]. Without E1B to inactivate p53, this H101 adenovirus cannot replicate and lyse normal cells where p53 is active. In addition, the deletion of a 78.3–85.8 µm gene segment in the *E3* region, which encodes the adenovirus death protein, may enhance the safety of the product [5]. However, H101 has limited efficacy as monotherapy in clinical practice. In order to increase its effectiveness, it is often combined with radiotherapy or chemotherapy.

Notch signaling plays a pivotal role in cellular differentiation, proliferation, and apoptosis [7]. The Notch proteins constitute a family of transmembrane proteins that form heterodimeric transmembrane receptors. Following ligand binding, the receptor catalyzes the cleavage of its own intracellular domain (ICN), which can then enter the nucleus to regulate target genes involved in regulating cell growth, cell differentiation and cell apoptosis [8], [9].

The Notch signaling pathway is disrupted in several malignancies, offering a potential target for therapeutic intervention. There is aberrant activation of Notch signaling in glioblastoma (GBM) cell lines and in human GBM-derived neurospheres. Inhibition of Notch signaling via the expression of a dominant negative form of the Notch co-activator, mastermind-like 1 (DN-MAML1) or the treatment of an γ-secretase inhibitor (GSI) MRK-003 resulted in a significant reduction in GBM cell growth *in vitro* and *in vivo* [10]. While there is abundant evidence that Notch signaling can stimulate the growth of wide range of tumors, the precise molecular mechanisms underlying alterations of this pathway during carcinogenesis are yet to be identified.

Notch is also critical in maintaining the ability of cancer stem cells (CSCs to self renew) (see reviews [11], [12], [13]). CSCs are a subpopulation of tumor cells that possess stem cell properties, including indefinite self-replication, pluripotency, and resistance to chemotherapeutic agents. CSCs are associated with tumor relapse and metastasis, and may also account for the ultimate failure of conventional cancer therapies [14], [15]. Cancer cures may require the complete elimination of the small cancer stem cell population of the tumor as well as of the non-CSC majority of cancer cells. Consequently, the idea of selectively targeting CSCs with novel therapeutics, e.g. those attacking Notch signal pathway, is gaining considerable interest.

Cervical cancer cell line Hela-S3 was deficient in p53, and preclinical studies demonstrated that Hela-S3 was very sensitive to H101 oncolytic treatment. We have previously shown that knockdown of the Notch 1 gene could inhibit the proliferation and growth of HeLa cells both *in vitro* and *in vivo* [16]. In this study, we test a dual therapeutic approach by simultaneously targeting p53 mutations and aberrant Notch signal activity in tumors. To accomplish this, we combined a Notch1 siRNA with the oncolytic adenovirus H101. It is assumed that H101 replication specifically lyses the bulk of cancer cells that are p53-inactive. At the same time, Notch1 siRNA targets both the Notch-pathway mutated tumors and the minority CSC population of the tumor. As a first step to prove this concept, in this communication we report the *in vitro* and *in vivo* therapeutic effects of H101/Notch1-siRNA combined therapy in HeLa-S3 tumor cells.

## Results

### Suppression of Notch1 by siRNA in Tumor Cells

Among the Notch family genes, Notch1 is the best validated target in malignancies, with the highest activating mutations identified in tumors. Our previous *in vitro* and *in vivo* studies demonstrated that knockdown of the Notch 1 gene inhibited the proliferation and growth of HeLa cells. We examined the expression of the Notch family genes in HeLa-S3 cells that lack the activity of p53. Using RT-PCR, we found that Notch1 was expressed in HeLa-S3 cells, while other three family members Notch2, Notch3, and Notch4 were barely detectable (**Fig. S1**). We thus focused on the well-validated Notch1 in this study.

**Figure 1:**
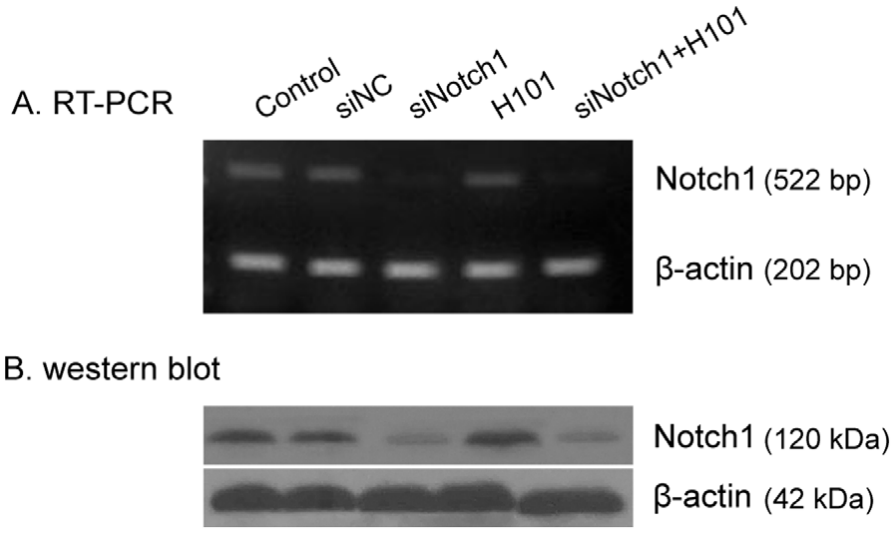
Notch1 gene knockdown by siRNA. A. Semi-quantitative RT-PCR analysis of Notch1gene transcripts in HeLa-S3 cells. The experiment was performed 48 hours after siNotch1 (100 nmol/l) transfection with or without H101 infection (multiplicity of infection (MOI)  = 100). β-actin was used as the internal control for normalization. B. Western Blot analysis of Notch1 protein in HeLa-S3 cells. The experiment was performed 72 hours after siNotch1 (100 nmol/l) transfection with or without H101 infection (multiplicity of infection (MOI)  = 100). Western bands were scanned and normalized over the internal control β-actin.

We then tested the suppression of Notch1 by its siRNA in HeLa-S3 tumor cells. Notch1-siRNA and control NC-siRNA were used to transfect HeLa-S3 cells, respectively, and the efficiency of siRNA on Notch1 expression was examined by RT–PCR and Western blot. As expected, no change in the abundance of Notch1 mRNA was detected in the H101 group. As compared with the NC-siRNA control, Notch1-siRNA, used either alone or with H101, suppressed Notch1 expression (**Fig. 1A**). Suppression of Notch1 by Nocth1-siRNA was also confirmed at the protein level by Western blot analysis. (**Fig. 1B**).

### Enhanced Cytotoxicity by the Combined Treatment of Notch1-siRNA and H101

**Figure 2:**
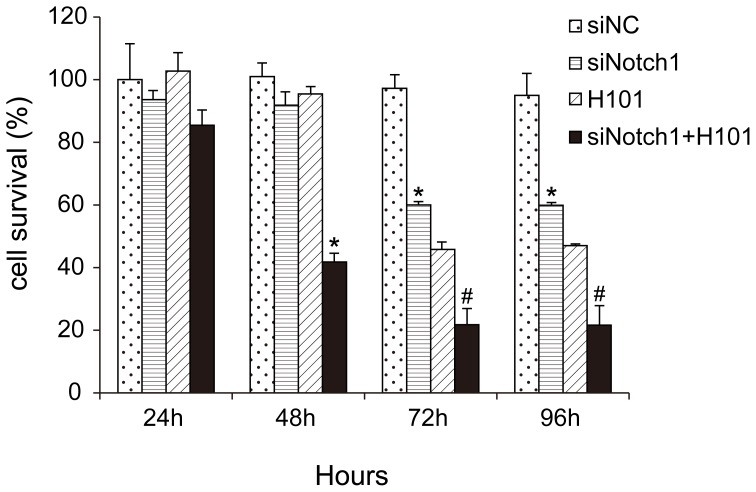
Cell proliferation of HeLa-S3 cells following the combined treatment of Notch1-siRNA and H101. Cell proliferation was measured by MTT assays 24, 48, 72, 96 hours after co-treatment of Notch1-siRNA (100 nmol/l) and 24, 48, 72 hours after H101 infection (multiplicity of infection (MOI)  = 100). All data are presented as means ± SD of three independent experiments. *p<0.05, #p<0.01 as compared with negative control.

Having established that Notch1-siRNA inhibited Notch1 expression, we used the MTT method to detect the effects of combined treatment of Nocth1-siRNA and H101 on cell growth (**Fig. 2**). In the siNotch1 group, a significant degree of proliferative inhibition was observed after 72 hours, suggesting that RNA interference blocked the endogenous Notch pathway and showed a delayed inhibition of cell growth. Similarly, inhibition of cell growth was detected 72 hours after cells were infected with H101 virus (MOI 100). In the combined treatment group, however, cell growth was significantly inhibited as early as 48 hours after the treatment, indicating an augmentation of growth inhibition. Similar data were also obtained in other tumor cell lines A549, OCM1 and VUP (Fig. S2). We used the normal cervical keratinocytes as the control. The data showed that the cell prolifercation was unaffected by H101 treatment (MOI = 100) in normal cervical keratinocytes (**Fig. S3**).

### Improved *in vivo* Antitumor Activity by the Combined Treatment of Notch1-siRNA and H101

To examine whether the enhanced *in vitro* tumor cytotoxicity could be translated into *in vivo* animal testing, we first initiated a pilot study by treating animals when HeLa-S3 tumor xenografts reached 200–300 mm^3^. Based on the data from this pilot study, we modified the protocol by initiating the treatment at an early stage when the average tumor volume reached about 100 mm^3^. At this stage, animals began to receive an intra-tumor injection of Notch1-siRNA, H101 or PBS every three days, for a total of four injections.

**Figure 3:**
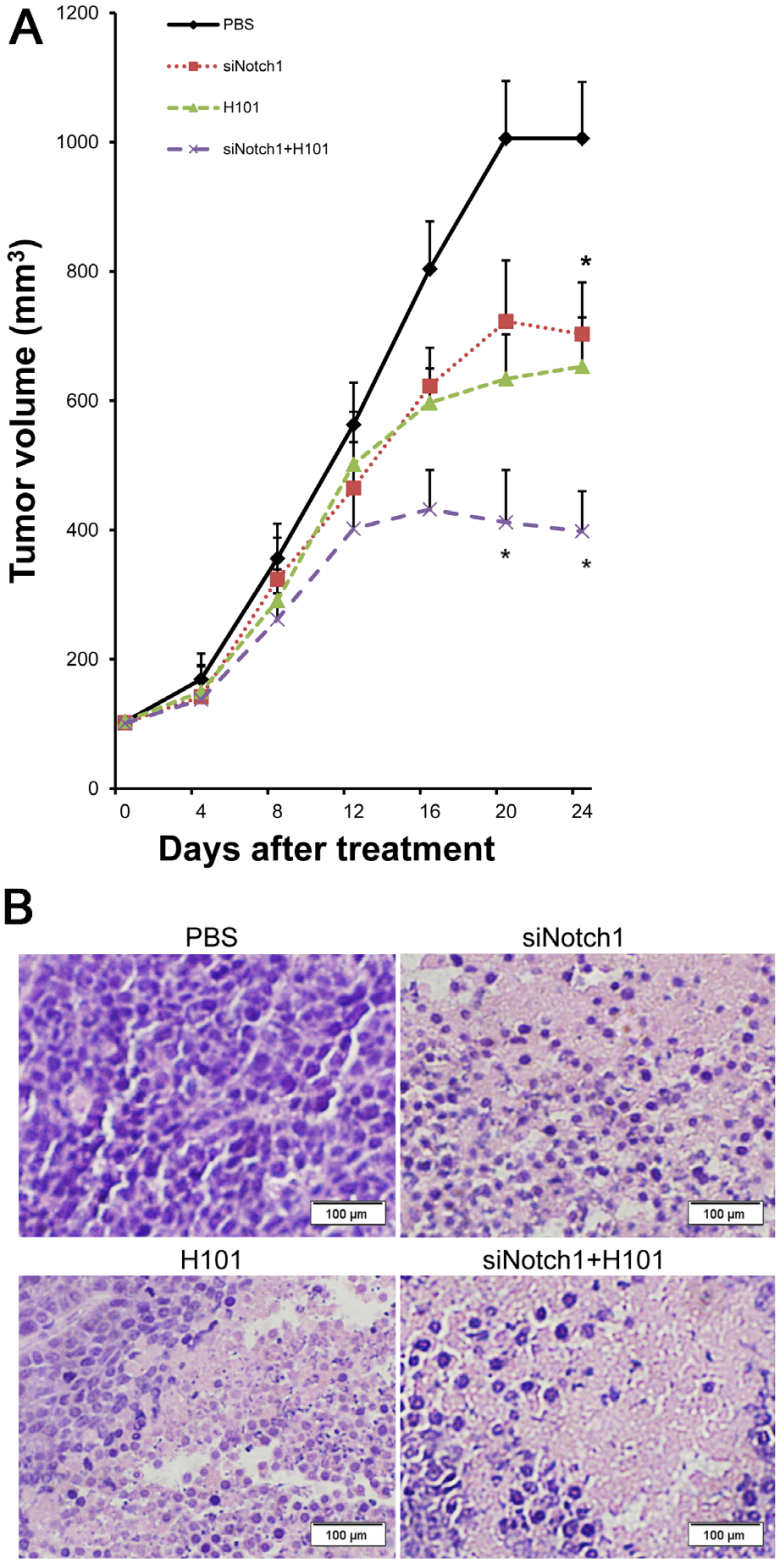
Antitumor activity of the cocktail treatment of Notch-siRNA and H101. A. HeLa-S3 xenograft tumors in nude mice. Average volume of subcutaneous tumors after treatment with H101(triangle), H101 plus Notch1-siRNA (cross), Notch1-siRNA (square), or PBS (rhombus). Values represent the means ± SD for five animals per group. (*p<0.05). B. Histological analysis of HeLa-S3 derived tumors. Original magnification: ×200.

As compared with the PBS control group, the Notch1-siRNA or H101 monotherapy showed similar inhibition of tumor growth. The Notch1- siRNA/H101 group, however, had an enhanced anti-tumor effect (**Fig. 3A**). Marked differences were seen in the degrees of inflammation and necrosis in the tumor specimens. Tumors from the H101-Notch1-siRNA treated group were more differentiated than those from the PBS treated group (**Fig. 3B**).

### Enhanced Apoptosis by the Combined Treatment of Notch1-siRNA and H101

**Figure 4:**
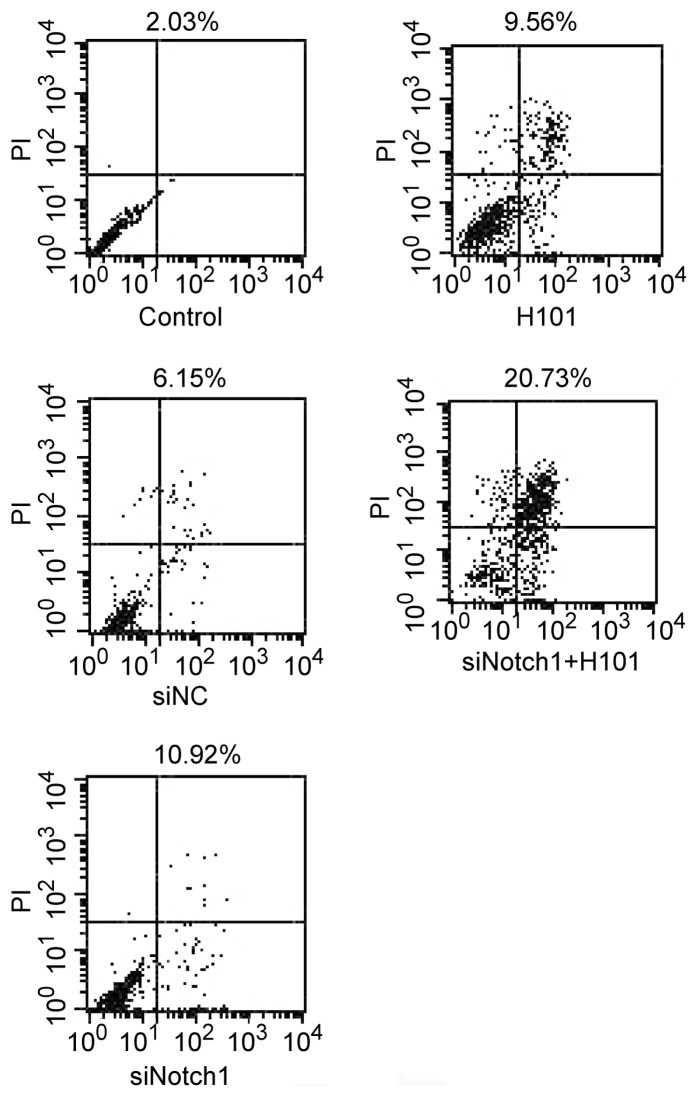
Apoptosis analysis of HeLa-S3 tumor cell induced by the H101- Notch1-siRNA treatment. HeLa-S3 cell was harvested 72 h after transfection, and Annexin V staining was used to analyze early-stage cell apoptosis.

To examine the mechanism underlying the augment of anti-tumor effect by the combined Notch1-siRNA/H101 treatment, cell apoptosis was measured using an Annexin V-FITC apoptosis kit and flow cytometric analysis 48 hours after the cells were transfected with Notch1-siRNA and H101. As seen in **Figure 4**, the combined treatment of Notch1-siRNA and H101 induced 20.7% apoptosis in treated cells as compared with 10.9% in Notch1-siRNA-treated cells and 9.6% in H101-treated cells. These data suggest an augment effect of Notch1-siRNA and H101 in inducing tumor apoptosis.

### Activation of Caspase-3 and Expression of Endogenous p53 and MDM2 after Combined Treatment of Notch1-siRNA and H101

**Figure 5:**
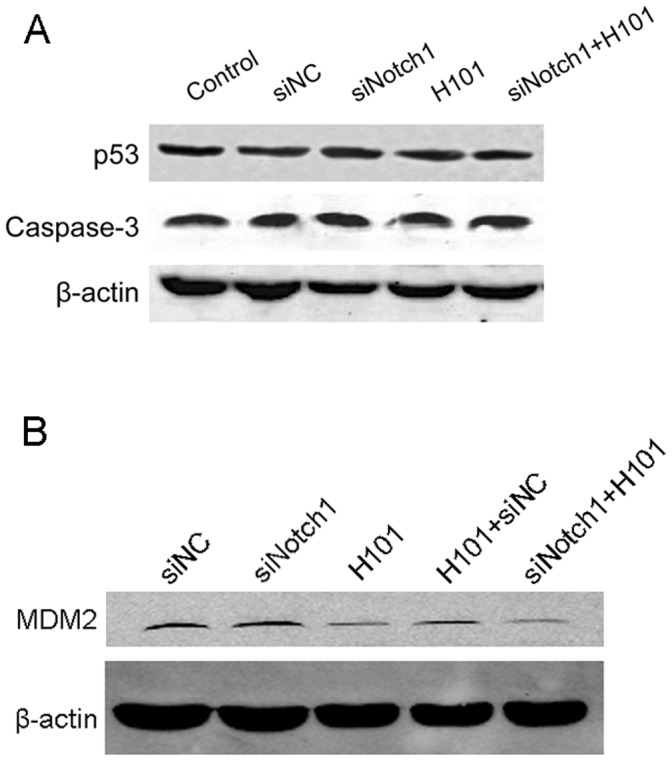
Expression of caspase-3 activation, p53 (A) and MDM2 (B) in HeLa-S3 tumor cells. Cells were transfected with the Notch1-siRNA and H101 for 72 hours, and total protein was analyzed by Western blot with specific antibodies.

We then used Western blot analysis to detect the activity of caspase-3, a critical component in cell apoptosis pathway. We found that the expression of caspase-3 did not change significantly among the treated groups (**Fig. 5A**, middle panel). Neither did we detect a significant amount of the cleaved caspase-3 (active form) in treated tumor cells. Using a more sensitive Caspase-3 Colorimetric Activity Assay Kit, we still could not detect a significant change of caspase-3 in the experimental groups (**Fig. S4**), suggesting that the combined therapy enhanced tumor apoptosis by a non-caspase-3 pathway.

We were also curious whether the combined Notch1-siRNA/H101 therapy would affect the expression of endogenous p53, an important component that affect H101 oncolysis and apoptosis. Three days after transfection with Notch1-siRNA and H101, HeLa-S3 cells were collected and total cellular protein was extracted. We found that the treatment of Notch1-siRNA, whether used alone or combined with H101, did not have significant effect on p53 protein level in treated cells (**Fig. 5A**, top panel).

We then used Western blot analysis to examine the expression of MDM2, a downstream target gene of p53. Three days after transfection with Notch1-siRNA and H101, both the H101 treatment and the combined H101/Notch-siRNA treatment significantly affected MDM2 protein expression in treated cells. Although the H101/siRNA combined therapy showed a slightly better effect than the H101/siNC control **(** **Fig. 5B** **)**, it seems that the downregulation of MDM2 was primarily derived from the H101 treatment. We also used the Western blot to measure the expression of p21, another p53 target gene. Similarly, we only detected a low level of p21 protein in the siNotch1 group (**Fig. S5**), indicating that the p53/p21 pathway may not be a significant factor in cell apoptosis induced by the combined therapy.

### Notch1-siRNA Enhanced H101 DNA Replication

We then focused on whether the Notch-siRNA alters viral replication in H101-infected tumors. Hexon protein is a component of the adenovirus capsid and is synthesized after cell infection. The synthesis of hexon protein marks the packaging of virus particles in the final replication stage. Thus, the amount of protein synthesized is considered to be a reliable indicator of viral replication.

**Figure 6:**
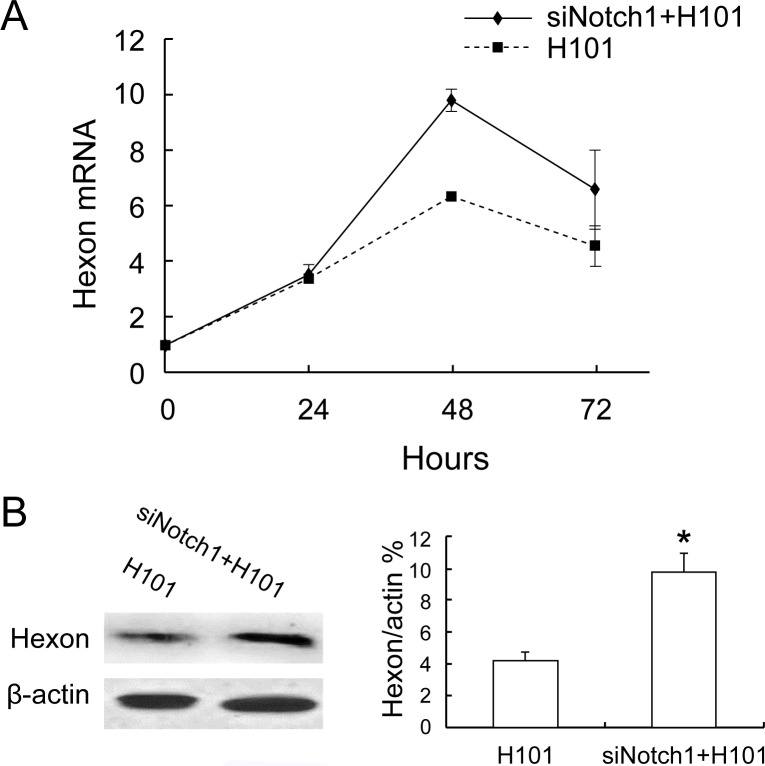
The effect of Notch1-siRNA on viral DNA replication in HeLa-S3 cells. A. Viral DNA replication was determined by real-time RT-PCR quantification of adenoviral late Hexon gene expression at 24, 48, and 72 hours after co-treatment, respectively. For comparison, the mRNA expression of Hexon at 24 hours in H101 group was arbitrarily set as 1, and β-actin was used as the internal control in the calculation. P<0.05: compared to Hexon mRNA expression of the H101 group. B. Western blot analysis of Hexon protein at 72 hours. Bar: average band density of quantified Hexon protein after normalization by the internal control β-actin. Protein expression of Hexon in H101 group was arbitrarily set as 1. *P<0.05: relative to Hexon protein expression in the H101-treated group.

To test whether Notch1-siRNA would affect H101 DNA replication, the expression of the late gene hexon was determined by real-time RT-PCR. We found that after Notch1-siRNA interference, the H101/Notch1-siRNA group had a significant increase of hexon mRNA expression compared with the H101 group (P<0.05, **Fig. 6A**). Similarly, Western blot analysis also showed an approximately two-fold increase in Hexon protein when the combination of Notch1-siRNA and H101 were used (P<0.05, **Fig. 6B**). Taken together, these data suggest that the silencing of Notch1 actually enhanced DNA synthesis of H101.

## Discussion

The Notch signaling pathway plays an important role in the regulation of cell growth and differentiation, tissue renewal, and cell homeostasis, and the pathway may be disregulated in several carcinomas [17], [18]. Notch1 antisense RNA treatment may lead to growth inhibition and even cell death if stably transfected in cervical cancer cells [19]. Notch signaling promotes cell survival, and the increment in Notch1 activity promotes tumor growth in lung adenocarcinoma when cultured under hypoxic conditions [20]. Synthetic triterpenoids inhibit growth and induce apoptosis in human glioblastoma and neuroblastoma cells through inhibition of Notch signaling [10], [21]. Notch1 directly regulates c-MYC and activates a feed-forward-loop transcriptional network promoting leukemic cell growth [22]. Down-regulation of Notch-1 contributes to cell growth inhibition and apoptosis in pancreatic cancer cells, including BxPC-3, HPAC, and PANC-1 [23]. Studies have demonstrated the existence of a novel intracellular mechanism for Notch1 regulation mediated by DDR1, in which deregulated DDR1 activation results in persistent autonomous activation of a Notch signaling and subsequent induction of Notch-dependent pro-survival neoplastics [24]. Our previous study demonstrates that blocking Notch1 signaling by RNA interference can induce growth inhibition in HeLa cells [16]. Furthermore, Notch is involved in the maintenance of self-renewal of cancer stem cells (CSCs) [11], [12], [13], contributing to tumor relapse, metastasis, and drug resistance [14], [15]. Thus, the Notch signaling pathway is a promising target for the development of new anti-cancer therapeutics.

There is also a functional link between Notch and p53 activities. Notch1 is a *p53* target gene involved in human keratinocyte tumor suppression through negative regulation of ROCK1/2 and MRCK kinases [25]. Notch1 is induced upon p53-dependent UVB exposure in skin cells [25], [26]. *p53* is a tumor suppressor gene that is often mutated in tumors [27]. Restoration of p53 expression in a human cancer cell line up-regulates the expression of Notch1 [28]. Interference of p53 activity by either the E6 protein of human papillomavirus or *p53* siRNA leads to a reduction of Notch1 at the transcriptional level in cervical cancer cells [29]. In this communication, we tested combined therapy of Notch1 siRNA with a p53-targeted oncolytic adenovirus H101, in order to target two common abnormalities in cancer cells. We demonstrated the augmented tumor-killing of the combined therapy both *in vitro* and *in vivo*, confirming the feasibility of this combined modality for future studies.

H101, which lacks E1B55-kDa, can specifically lyse tumor cells. However, H101 has limited potential to eradicate tumors when used as monotherapy. Thus, H101 is often used in combination with traditional modalities, such as chemotherapy. In this communication, we studied the antitumor efficacy of H101 in conjunction with siRNA to Notch1. As demonstrated in **Figure 1**, Notch1-siRNA efficiently inhibited the expression of Notch1 mRNA and protein. Interestingly, the RNAi activity was not affected by the infection of oncolytic adenovirus H101. The combined action of Notch1 knockdown and H101 oncolysis significantly inhibited tumor growth *in vitro*, suggesting an additional effect of the combined tumor therapy. In the animal studies, we used direct intratumoral injections of high concentrations of Notch1-siRNA to increase the efficiency of intracellular transport of siRNA in the animal models. Direct intratumoral injection of H101 suspension also showed significant inhibition of growth in the nude mouse tumor model. Compared with monotherapy with either agent, the combined treatment of Notch-siRNA and H101 showed better tumor inhibition and prolonged the survival of animals bearing the tumor.

We also tested this combined therapy in other three tumor cell lines that had different status of p53 mutations, including lung cancer cells (A549) and uveal melanoma cells (OCM1 and VUP). Both OCM1 and VUP cell lines contain a common mutation (C. 797G>A, P. Gly133Glu) in the exon 7 of p53 [30], thus serving as a ideal therapeutic target for H101. However, we also noticed that A549, a cell line known to harbor a wild type p53, also responded to the H101 monotherapy (Fig. S2). Other two groups [31], [32] also reported that a second oncolytic adenovirus ONYX-015 was also able to replicate in A549 cells. Theoretically, the mutant virus with the deleted E1B, like H101, is able to replicate only in p53-defifcient cells. The molecular basis for such p53 status-independent effect of H101 in certain tumor cells, like A549, remains to be determined [31].

We also examined the cytotoxic effect of this combined therapy on cancer stem cells (CSCs). We first infected HeLa S3 cells with H101 and/or siNotch1. After 24 hours, cancer cells were cultured in CSC sphere culture medium and CSC spear numbers were recorded [24]. Because CSCs in HeLa-S3 cells were very low, we observed only a few CSC spears in control cells, but none in the group treated with the combined therapy. An ongoing study is under the way to isolate CSCs first and then to treat them with H101 and/or siNotch1.

The mechanism underlying the additive effect of the combined therapy remains uncharacterized. In tumor cells treated by the combined Notch1-siRNA and H101, we found an enhanced induction of apoptosis in HeLa-S3 cells, but we did not detect significant alteration of caspase-3 or activated caspase-3 expression. Neither Notch1-siRNA nor H101 appears to induce apoptosis by a non-caspase-3 pathway, and neither agent alters p53 expression. We also examined the expression of MDM2 and p21, which are the downstream targets of p53. MDM2 is an E3 ubiquitin ligase that targets p53 for ubiquitination and degradation. Both the MDM2 C-terminal region including the RING finger and the acidic domain are essential for p53 ubiquitination [33], [34]. MDM2 ablation in mice results in early embryonic lethality due to elevated levels of p53-induced apoptosis, and this phenotype is reversed by the simultaneous deletion of p53, demonstrating the importance of MDM2 in suppressing p53 [35], [36]. In our study, we did not detect a significant alteration of MDM2 from the Notch1-siRNA therapy. Similarly, no significant changes were noticed for p21, a second p53 target gene, in the combined therapy group.

Numerous studies have shown that the efficiency of adenovirus infection is related to the tumor cell surface receptor CAR. However, the expression of CAR in some tumor cells is relatively low. Extensive studies have attempted to alter viral tropism to increase infection rates and improve the anti-cancer effect [37], [38]. Adenovirus group C that lacks E1B 55-kDa protein is replicable, and its replication efficiency is also related to the expression of p53 in host cells [39]. E1B55-kDa protein affects oncolysis of adenovirus by several mechanisms, including the inhibition of p53 and pRB expression, regulation of the RNA output, turning-off of host protein synthesis, release of E2F, and inhibition of apoptosis [40], [41]. Interestingly, we found that Notch1 knock-down increased the amplification of the adenovirus as measured by late gene Hexon protein. Thus, promotion of viral replication by Notch1-siRNA may partially explain the increased antitumor efficacy in our combined therapeutic approach.

In an attempt to improve H101 anti-tumor efficacy, we previously demonstrated that H101 therapy was potentiated by concomitant use of a *Bcl2* siRNA. In mice bearing human xenograft tumors, all treated animals survived, and that some animals were tumor-free following the combined therapy [42]. In this extended study, we targeted the Notch signaling pathway that plays an important role in tumorigenesis by maintaining the activity of self-renewal of cancer stem cells. We hypothesized that interference of Notch signaling may inhibit tumor formation and progression by cutting off the source for cancer stem cells, therefore enhancing the therapeutic efficacy of oncolytic H101. It would be interesting in future studies to examine whether these strategies can be combined to offer a “three punch" approach by targeting p53 deficiency, *Bcl2* overexpression, and cancer stem cell.

In summary, this study provides support for the combined use of an oncolytic adenovirus and Notch1-siRNA as a promising approach in cancer gene therapy. We demonstrated an anticancer augmentation of the combined therapy of Notch1-siRNA and H101. Future studies will combine two therapies as a single adenoviral agent by integrating Notch1-siRNA into the H101 viral backbone. In addition, it will be interesting to examine whether the strategy used here would be more potent to target cancer stem cells (CSCs), particularly those CSCs cultured from clinical surgery or biopsy tumors.

## Materials and Methods

### Cell Culture and Recombinant Adenovirus H101

Cervical cancer cell line HeLa-S3 is deficient in p53, and preclinical studies demonstrated that HeLa-S3 was very sensitive to H101 oncolytic treatment [43]. Our previous *in vitro* and *in vivo* studies also demonstrated that knockdown of the Notch 1 gene inhibited the proliferation and growth of HeLa cells [16]. We thus tested our combined p53 and Notch therapy in this p53-deficent cervical cancer cell line. In addition, three tumor cell lines with different status of p53 mutations, including lung cancer A549 (wild type p53), uveal melanoma OCM1 and VUP (mutated p53), were also used for the study.

Tumor cell lines HeLa-S3 (cervical cancer) and A549 (lung cancer cells) were obtained from American Type Culture Collection (Manassas, VA, USA). OCM1 and VUP (uveal melanoma were kindly provided by Professor John F. Marshall (Tumor Biology Laboratory, Cancer Research UK Clinical Center, John Vane Science Centre, London, UK) [44]. HeLa-S3 cells were cultured at 37°C under 5% CO_2_ in Dulbecco’s modified Eagle’s medium (Gibco, Carlsbad, CA, USA) supplemented with 10% newborn calf serum (PAA Laboratories GmbH, Cölbe, Germany). Recombinant adenovirus H101 was kindly provided as a gift by Shanghai Sunway Biotech (Sunwaybio, Shanghai, China) and was maintained under conditions recommended by the manufacturer.

### RNA Interference

The Notch1 siRNA duplexes were produced by Shanghai Genepharma Co. Inc. (Genepharma, Shanghai, China) against human Notch1 (5′-AAG GUG UCU UCC AGA UCC UGA-3′). Scrambled fluorescent-labeled siRNA (5′-AAA UGU GUG UAC GUC UCC UCC-3′) (siNC) [30] was also designed and used as the negative control in the study.

### Co-treatment of Tumor Cells with *Notch1* siRNA and Oncolytic Adenovirus H101

HeLa-S3 cells at 30–50% confluence in 24-well plates were transfected with 80 nmol/l Notch1 siRNA using Lipofectamine 2000 following the manufacturer’s instructions (Invitrogen, Carlsbad, CA, USA). After overnight incubation, cells were infected with H101 at a multiplicity of infection of 100 MOI. Control groups included cells that were transfected with siNC or PBS.

### RNA Extraction and Reverse Transcription–PCR Analysis

Total RNA was isolated from treated cells using Trizol reagent (Invitrogen) following the protocol provided by the manufacturer. Total RNA (1µg) was reverse-transcribed into cDNA using M-MLV reverse transcriptase (Invitrogen) [45], [46]. The cDNA was used to amplify the Notch1 fragments. For normalization of RNA, the housekeeping gene β-actin was also amplified from each sample. The primer sequences were as follows: Notch1 (forward primer: 5′-TTCCCTGAGGGCTTCAAAGT-3′, reverse primer: 5′-CCCGCTACTCACGCTCTGAT-3′, 522 bp), Notch2 (forward primer: 5′-TTGCTGTTGCTGTTGTCATCA-3′, reverse primer: 5′-AAGGTGCTGCTGTGTCCAT-3′, 338 bp), Notch3 (forward primer: 5′-CTGTCTTGCTGCTGGTCATTC-3′, reverse primer: 5′-GTGTCATCTGCCTCATCCTCT-3′, 496 bp), Notch4 (forward primer: 5′-TGCTGCTGCTGCTGCTAT-3′, reverse primer: 5′-CTGCTCACCTGTCCATCCA-3′, 428 bp ), GAPDH (forward primer: 5′-GGATTTGGTGGTATTGGG-3′, reverse primer: 5′-GGAAGATGGTGATGGGATT-3′, 428 bp) and β-actin (forward primer: 5′-CCTTCCTGGGCATGGAGTCCT-3′, reverse primer: 5′-GGAGCAATGATCTTGATCTT-3′, 202 bp). RT-PCR amplification was performed using the following conditions: 95°C for 5 min, 1 cycle; 94°C for 45 sec, 56°C for 45 sec and 72°C for 45 sec, 30 cycles. After amplification, 10 µl of PCR product was run on a 1.5% agarose gel and visualized by ethidium bromide staining.

Quantitative real-time RT-PCR amplification was carried out using Real-Time MIX (SYBR Premix Ex TaqTM, TaKaRa, Tokyo, Japan). Specifically, total RNA was extracted by Trizol reagent (Invitrogen), and cDNA was synthesized with RNA reverse transcriptase. The C_T_ (threshold cycle) value of *Hexon* was quantitated by Q-PCR in triplicate using an ABI Prism 7900 HT sequence detector (AB Applied Biosciences, CA, USA) following the manufacturer’s protocol and was normalized over the C_T_ of the β*-actin* control [47], [48].

### Western Blot Analysis

Cells were harvested at the indicated time, and proteins were separated by sodium dodecyl sulfatepolyacrylamide gel electrophoresis in 10% SDS–polyacrylamide Tris–glycine gels for protein expression. The immunoblotting was performed with the Notch1 (Epitomics, CA, USA), p53 (Cell Signaling Technology, Inc., Danvers, MA, USA), Caspase 3 (Thermo Fisher Scientific, Loughborough, UK), Hexon (Abcam, Cambriadge, UK) and mouse β-actin (Sigma-Aldrich, St. Louis, MO, USA), followed by detection with a horseradish peroxidase–conjugated secondary antibody [42].

### Caspase-3 Activity Assay

Cells were seeded at 2×10^5^ cells per well in flat-bottomed 6-well plates. At the end of the incubation time, the cells were harvested and the Caspase-3 Colorimetric Activity Assay Kit was used to detect the activity of caspase-3 [49].

### MTT Assay

The 3-(4, 5-dimethylthiazol-2-yl)-2,5-diphenyl-2H-tetrazolium bromide (MTT) assay was performed to assess the effect of H101 in combination with RNA interference on cell proliferation [42]. Cells were seeded in 96-well plates at a concentration of 5×10^3^ cells/well. At the end of the incubation, 20 µl of 5 mg/ml MTT (Sigma-Aldrich) in PBS was added to each well. Each experiment was repeated five times. Absorbance was measured after a further 4 h incubation at 37°C with a solution of MTT (0.33 mg/ml) that contained 12.5µM Dimethyl sulfoxide (DMSO). The absorbance was measured on a spectrophotometer microplate reader at a wavelength of 492 nm.

### Apoptosis Analysis

Apoptosis was determined by dual staining with annexin-V-fluorescein isothiocyanate and propidium iodide and analyzed by flow cytometry [42]. Cells were prepared according to the manufacturer’s instruction provided in the Annexin V- FITC apoptosis kit (BD Biosciences, San Diego, CA, USA). Apoptosis was quantified on a fluorescence-activated cell sorter (Becton Dickinson, Sunnyvale, CA), and data from 10,000 events were collected for further analysis.

### Tumor Xenograft Model in Nude Mice

Animal experiments were performed in accordance with institutional guidelines for animal care by Jiao Tong University. Specifically, HeLa-S3 cell tumor xenografts were established by s.c. injection of 1×10^6^ cells into the right flank of 4–6-week-old male athymic nude mice. Based on the data from a pilot study, we initiated an early treatment when the tumor volume reached about 100 mm^3^ (volume = length×width^2^×0.5). Animals were randomly assigned into four groups. The Notch1-siRNA plus H101 group received intratumoral injections of 10 µg Notch1-siRNA on day 1, 4, 8, 11, 15, and H101 adenovirus at 1×10^8^ plague-forming units on day 2, 5, 9, 12, and 16. The Notch1-siRNA group received five intratumoral injections of 10 µg Notch1-siRNA. The H101 adenovirus group received five intratumoral injections of H101. The control group mice received five injections of PBS. The tumor size was measured by vernier calipers every 4 days. Mice from each group were selected randomly and killed on day 7 after treatment for hematoxylin-eosin staining.

Specimens were dehydrated in ethanol series (80, 85, 80, 90, 95 and 100%), embedded in paraffin, and cut into 5 µm-thick sections. Sections were then deparaffinized, stained with hematoxylin/eosin (H–E) following standard protocol, and observed using a light microscope.

### Statistical Analysis

All experiments were performed in triplicate, and the data were expressed as mean ± SD. The data were analyzed with one-way analysis of variance (ANOVA), and results were considered statistically significant at P≤0.05.

## Supporting Information

Figure S1 **The expression of Notchs 1, 2, 3 and 4 in HeLa-S3 cells.** Semi-quantitative RT-PCR analysis of Notch 1, Notch 2, Notch 3 Notch 4 and GAPDH gene transcripts in HeLa-S3 cells.
(TIF)

Figure S2 **Cell proliferation of A549 (A), OCM-1 (B) and VUP (C) cells following the combined treatment of Notch1-siRNA and H101.** Cell proliferation was measured by MTT assays 24, 48, 72, 96 hours after co-treatment of Notch1-siRNA (100 nmol/l) and 24, 48, 72 hours after H101 infection (multiplicity of infection (MOI)  = 100). All data are presented as means ± SD of three independent experiments. *p<0.05, # p<0.01 as compared with negative control.
(TIF)

Figure S3 **Cell proliferation of normal cervical keratinocytes cells following the combined treatment of Notch1-siRNA and H101.** Cell proliferation was measured by MTT assays 72 hours after co-treatment of Notch1-siRNA (100 nmol/l) and 48 hours after H101 infection (multiplicity of infection (MOI)  = 100). All data are presented as means ± SD of three independent experiments.
(TIF)

Figure S4 **The activity of caspase-3 in Hela-S3 tumor cells.** Cells were transfected with the Notch1-siRNA and H101 for 72 hours, and total protein was analyzed by the Caspase-3 Colorimertric Activity Assay Kit.
(TIF)

Figure S5 **Expression of p21 in HeLa-S3 tumor cells.** Cells were transfected with the Notch1-siRNA and H101 for 72 hours, and total protein was analyzed by Western blot with specific antibodies.
(TIF)

Method S1 **The sphere assay.**
(DOCX)
