# Supplementary figures and images for: A Novel Anticancer Therapy That Simultaneously Targets Aberrant p53 and Notch Activities in Tumors
Source: PLoS One. 2012 Oct 10;7(10):e46627. doi: 10.1371/journal.pone.0046627 (PMC3468572; doi:10.1371/journal.pone.0046627)

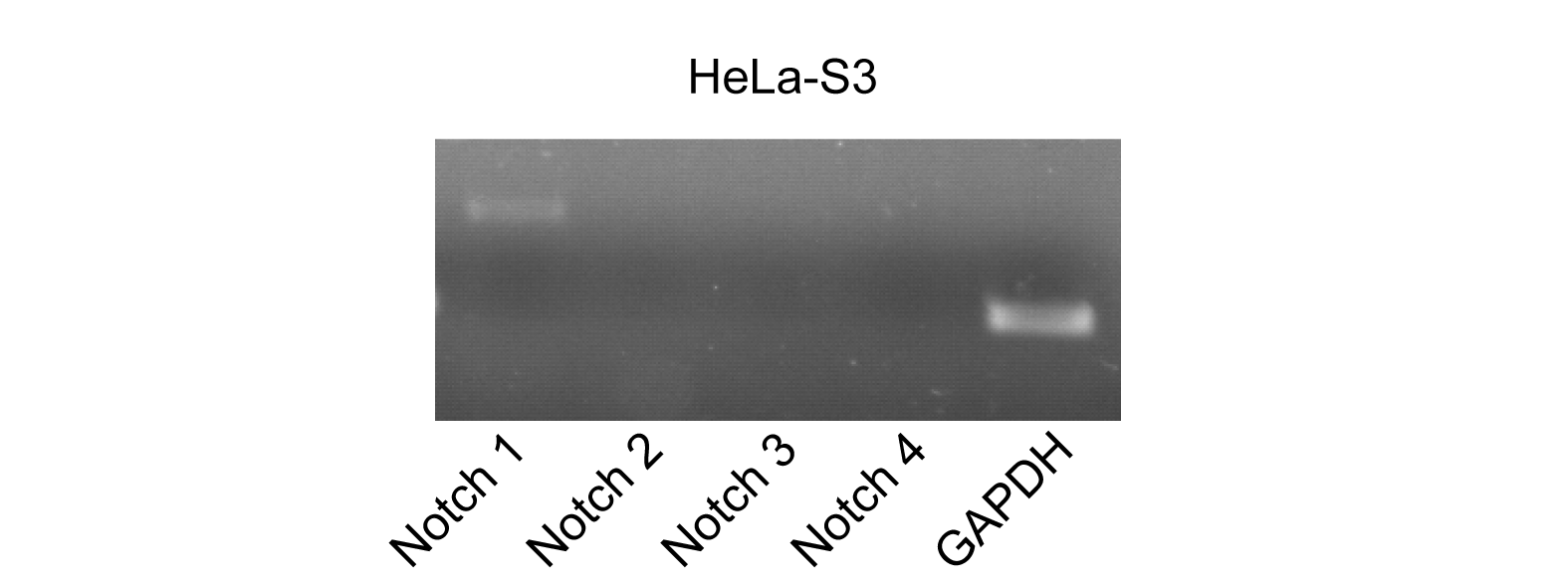

Supplement: Figure S1 — The expression of Notchs 1, 2, 3 and 4 in HeLa-S3 cells. Semi-quantitative RT-PCR analysis of Notch 1, Notch 2, Notch 3 Notch 4 and GAPDH gene transcripts in HeLa-S3 cells. (TIF) [file pone.0046627.s001.tif]

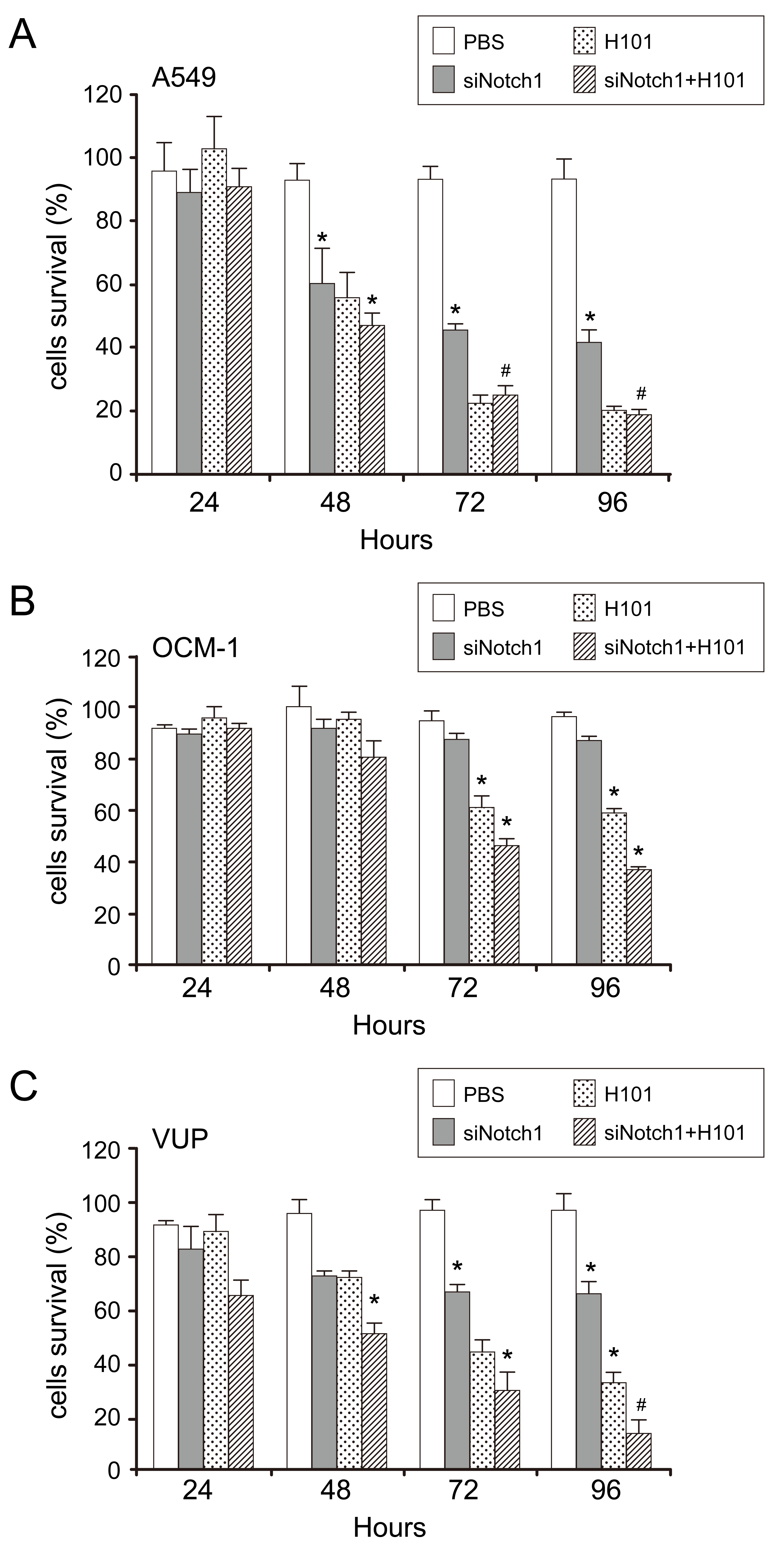

Supplement: Figure S2 — Cell proliferation of A549 (A), OCM-1 (B) and VUP (C) cells following the combined treatment of Notch1-siRNA and H101. Cell proliferation was measured by MTT assays 24, 48, 72, 96 hours after co-treatment of Notch1-siRNA (100 nmol/l) and 24, 48, 72 hours after H101 infection (multiplicity of infection (MOI) = 100). All data are presented as means ± SD of three independent experiments. *p<0.05, # p<0.01 as compared with negative control. (TIF) [file pone.0046627.s002.tif]

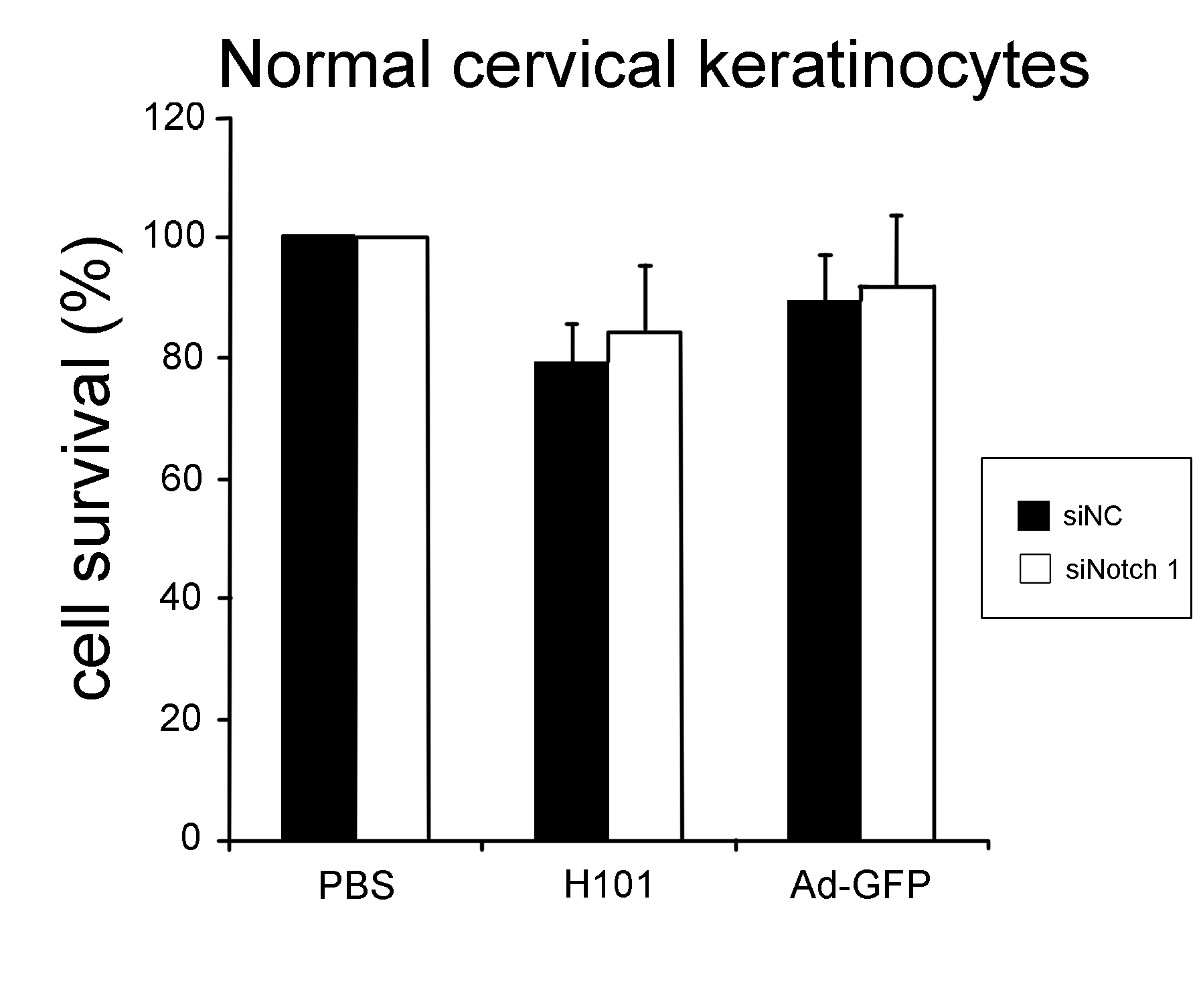

Supplement: Figure S3 — Cell proliferation of normal cervical keratinocytes cells following the combined treatment of Notch1-siRNA and H101. Cell proliferation was measured by MTT assays 72 hours after co-treatment of Notch1-siRNA (100 nmol/l) and 48 hours after H101 infection (multiplicity of infection (MOI) = 100). All data are presented as means ± SD of three independent experiments. (TIF) [file pone.0046627.s003.tif]

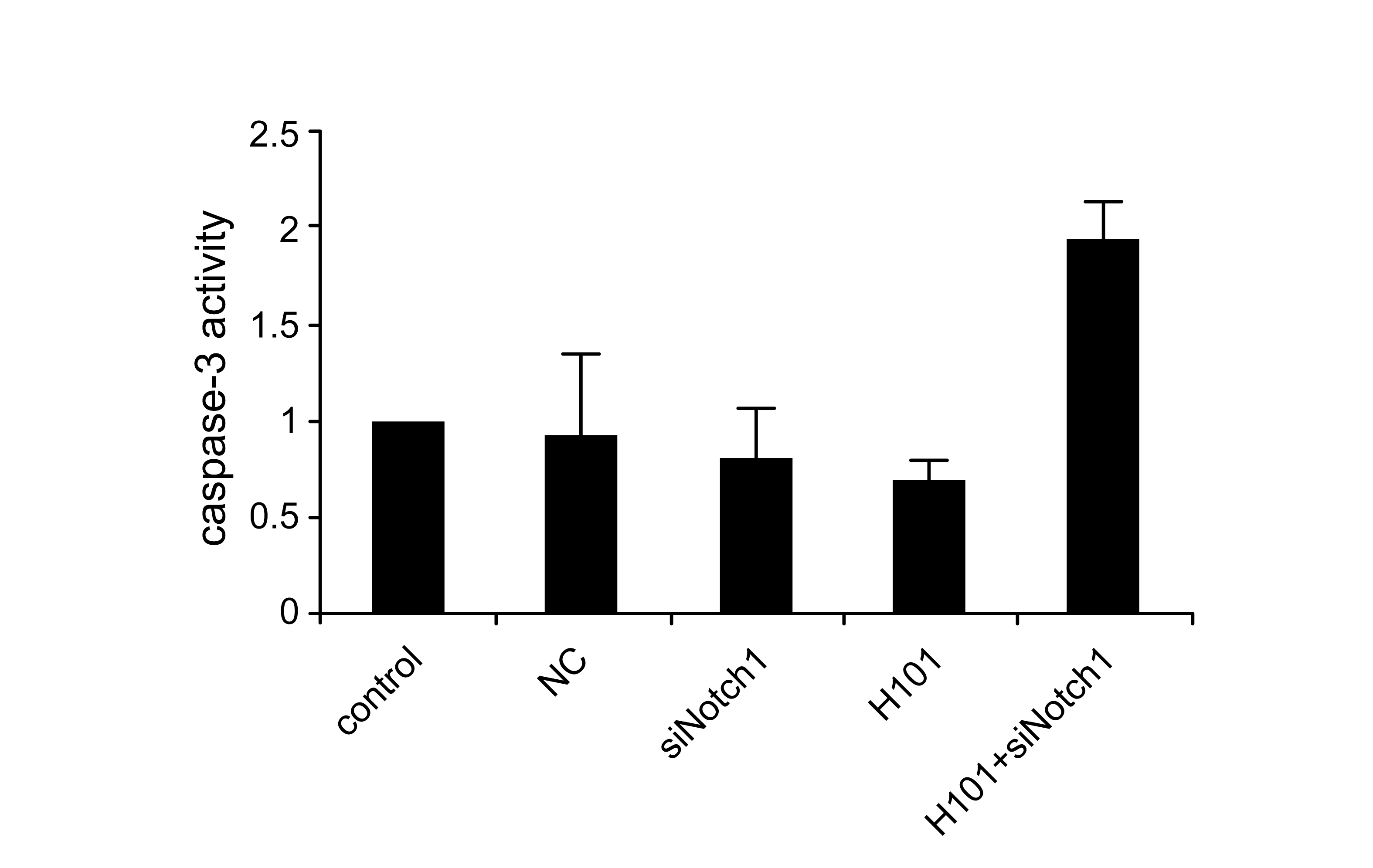

Supplement: Figure S4 — The activity of caspase-3 in Hela-S3 tumor cells. Cells were transfected with the Notch1-siRNA and H101 for 72 hours, and total protein was analyzed by the Caspase-3 Colorimertric Activity Assay Kit. (TIF) [file pone.0046627.s004.tif]

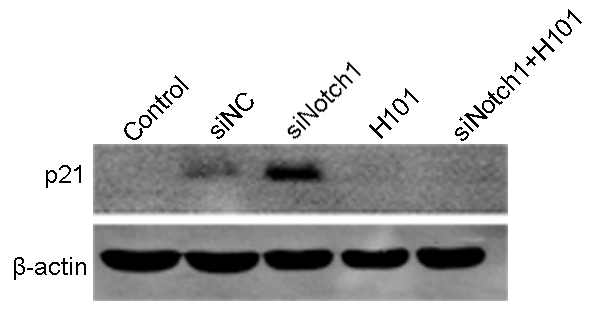

Supplement: Figure S5 — Expression of p21 in HeLa-S3 tumor cells. Cells were transfected with the Notch1-siRNA and H101 for 72 hours, and total protein was analyzed by Western blot with specific antibodies. (TIF) [file pone.0046627.s005.tif]
