# Supplementary material for: A Novel Anticancer Therapy That Simultaneously Targets Aberrant p53 and Notch Activities in Tumors
Source: PLoS One. 2012 Oct 10;7(10):e46627. doi: 10.1371/journal.pone.0046627 (PMC3468572; doi:10.1371/journal.pone.0046627)
Supplement: Method S1 — The sphere assay. (DOCX) [file pone.0046627.s006.docx]

**Method S1. The sphere assay.**

Cervical cancer cell line HeLa cells were infected with H101 at a multiplicity of infection of 100 MOI. After 24 hours, 1×10^4^ cancer cells were seeded into a T25 flask with 8ml of sphere culture medium. An additional 4ml of sphere culture medium was added to the culture on day 4 and the culture continued for another 4–5 days.
